# A mechanism for MEX-5-driven disassembly of PGL-3/RNA condensates in vitro

**DOI:** 10.1073/pnas.2412218122

**Published:** 2025-05-12

**Authors:** Natasha S. Lewis, Silja Zedlitz, Hannes Ausserwöger, Patrick M. McCall, Lars Hubatsch, Marco Nousch, Martine Ruer-Gruß, Carsten Hoege, Frank Jülicher, Christian R. Eckmann, Tuomas P. J. Knowles, Anthony A. Hyman

**Affiliations:** ^a^Max Planck Institute of Molecular Cell Biology and Genetics, Dresden 01307, Germany; ^b^Max Planck School Matter to Life, Heidelberg 69120, Germany; ^c^Centre for Misfolding Diseases, Yusuf Hamied Department of Chemistry, University of Cambridge, Cambridge CB2 1EW, United Kingdom; ^d^Center for Systems Biology, Dresden 01307, Germany; ^e^Cluster of Excellence Physics of Life, Technische Universität, Dresden 01307, Germany; ^f^Leibniz Institute of Polymer Research, Dresden 01069, Germany; ^g^Max Planck Institute for the Physics of Complex Systems, Dresden 01187, Germany; ^h^Institute of Biology, Martin Luther University Halle-Wittenberg, Halle, Saale 06120, Germany; ^i^Cavendish Laboratory, Department of Physics, University of Cambridge, Cambridge CB3 0HE, United Kingdom

**Keywords:** phase-separation, *C. elegans*, polarity

## Abstract

MEX-5 regulates the formation and dissolution of P granules in *Caenorhabditis elegans* embryos, yet the thermodynamic basis of its activity remains unclear. Here, using a time-resolved in vitro reconstitution system, we show that MEX-5 dissolves preassembled liquid-like PGL-3/RNA condensates by altering RNA availability and shifting the phase boundary. We develop a microfluidic assay to systematically analyze how MEX-5 influences phase separation. By measuring the contribution of PGL-3 to phase separation, we show that MEX-5 reduces the free energy of PGL-3, shifting the equilibrium toward dissolution. Our findings provide a quantitative framework for understanding how RNA-binding proteins modulate condensate stability and demonstrate the power of microfluidics in precisely mapping phase transitions.

## Significance

Cells use biomolecular condensates to organize biochemical reactions without membranes, but how these structures form and dissolve remains poorly understood. In early *Caenorhabditis elegans* embryos, P granules must disassemble to ensure proper cell fate, a process linked to the RNA-binding protein MEX-5. Using a reconstituted system, we show that MEX-5 dissolves P granules by altering RNA availability, shifting the phase boundary, and reducing the free energy of condensate formation. Our findings provide quantitative insights which help to comprehend how cells regulate condensate stability, with implications for understanding phase separation in development and disease.

P granules are an example of membrane-less organelles enriched in the germ lineage of the nematode *Caenorhabditis elegans* (1, 2) and consist of numerous proteins and RNA (3–6). These proteins include RGG-rich proteins like PGLs (7, 8), RNA helicases like GLHs (9), and intrinsically disordered proteins like MEG-3/4 (10–12). In oocytes, P granules are symmetrically distributed in the cytosol and on the nuclear pores, while after fertilization and concomitant with polarization, cytosolic P granules segregate to one end of the one-cell stage embryo. It has been proposed that segregation of P granules is required for formation of the future germline (13) and controlled by the embryo polarity machinery. At the one-cell stage, the cortex separates into two domains, the establishment of which results in formation of a cytosolic MEX-5 gradient (Movie 1), and this gradient of MEX-5 has been shown to be required for P granule segregation (14–17).

The mechanism of P granule segregation is thought to be linked to the fact that P granules are liquids that form by phase separation from the cytoplasm. This suggests that P granules segregate by position-dependent phase separation, with phase separation favored at the posterior and disfavored at the anterior side of the cell (18). Because the MEX-5 gradient is opposite to the gradient of P granule segregation, it has also been proposed that P granule segregation results from MEX-5 activities that suppress P granule condensation in the anterior, allowing P granules to form by phase separation in the posterior (18, 19). Clues for how MEX-5 may suppress P granule condensation come from the observation that MEX-5 has high affinity, but low specificity, for RNA. Because P granule assembly also requires RNA, MEX-5 might outcompete P granule proteins for RNA binding, thus suppressing P granule formation by competing for RNA at the anterior pole (19). However, the mechanism by which MEX-5 interferes with P granules and leads to their dissolution is not well understood.

Here, we establish an in vitro assay to investigate the direct role of MEX-5 in regulating P granule formation. We show that MEX-5 dissolves preassembled liquid-like PGL-3/poly-rU RNA condensates and shifts the threshold for condensate formation to higher concentrations of both RNA and PGL-3. To quantify these effects, we employ a high-throughput microfluidic platform that allows systematic mapping of the phase boundary and precise measurement of dilute-phase PGL-3 concentrations. This approach reveals that MEX-5 reduces the free energy contribution of PGL-3 to phase separation, suggesting that MEX-5 modulates condensate stability by altering RNA availability and valency. Together, our findings provide mechanistic insight into how MEX-5 controls the dissolution of minimal P granule condensates and demonstrate the power of microfluidics in dissecting biomolecular phase behavior with high precision.

## Results

### RNA Lowers the Saturation Concentration (*c*_sat_) of Constitutive P Granule Proteins, PGL-1 and PGL-3, in *C. elegans*.

**Fig. 1.**
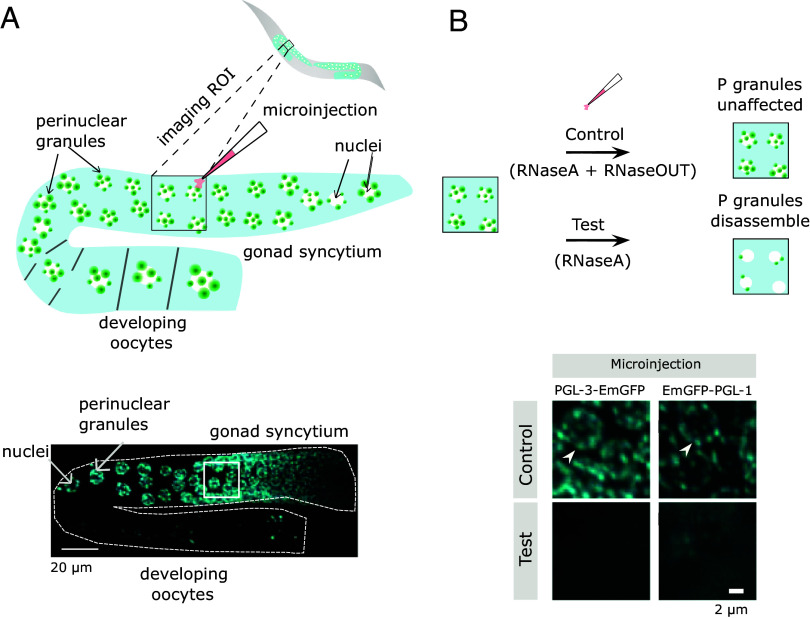
RNA lowers the saturation concentration of constitutive P granule proteins, PGL-1 and PGL-3, in *Caenorhabditis elegans*. (*A*) Illustration of gonadal injections of an adult worm (*Top*) and maximum intensity projections of confocal z-slices of syncytial germ cells expressing PGL-3-mEGFP (*Bottom*). The imaging ROI is immediately upstream or downstream from the point of injection. (*B*) Illustration (*Top*) and maximum intensity projections of confocal z-slices of PGL-3-mEGFP or mEGFP-PGL-1 condensates (*Bottom*). Test injection contains RNaseA (range 5 to 15 μg/ml) and control injection with RNaseA (range 5 to 15 μg/ml) preincubated with RNaseOUT. Images were taken 15 min after injection in test and control worms (n = 3). We were unable to follow the injected oocytes into embryogenesis because the injections resulted in oocyte arrest in test condition. Arrows point at the perinuclear PGL-3-mEGFP, mEGFP-PGL-1 condensates.

First, we wanted to confirm the role of RNA in P granule assembly in vivo. To do this, we degraded RNA in the gonad by microinjecting RNaseA, an endonuclease that cleaves single-stranded RNA (20), into the gonad of the worms (Fig. 1*A*). Microinjecting RNaseA resulted in the loss of the perinuclear PGL-1 and PGL-3 condensates in test worms (Movie 2). In contrast, control worms microinjected with the same concentration of RNaseA but mixed with RNaseOUT, an RNase inhibitor, maintained the perinuclear assemblies of the respective P granule proteins (Fig. 1*B*). Although the microinjection volumes cannot be normalized to the gonad volume, these qualitative experiments indicate that RNA is required for the integrity of P granules in the gonad. Similar experiments have shown a requirement for RNA in assembling stress granules (21).

### RNA Sequence and Concentration Regulate the Saturation Concentration and the Properties of In Vitro PGL-3 Condensates.

**Fig. 2.**
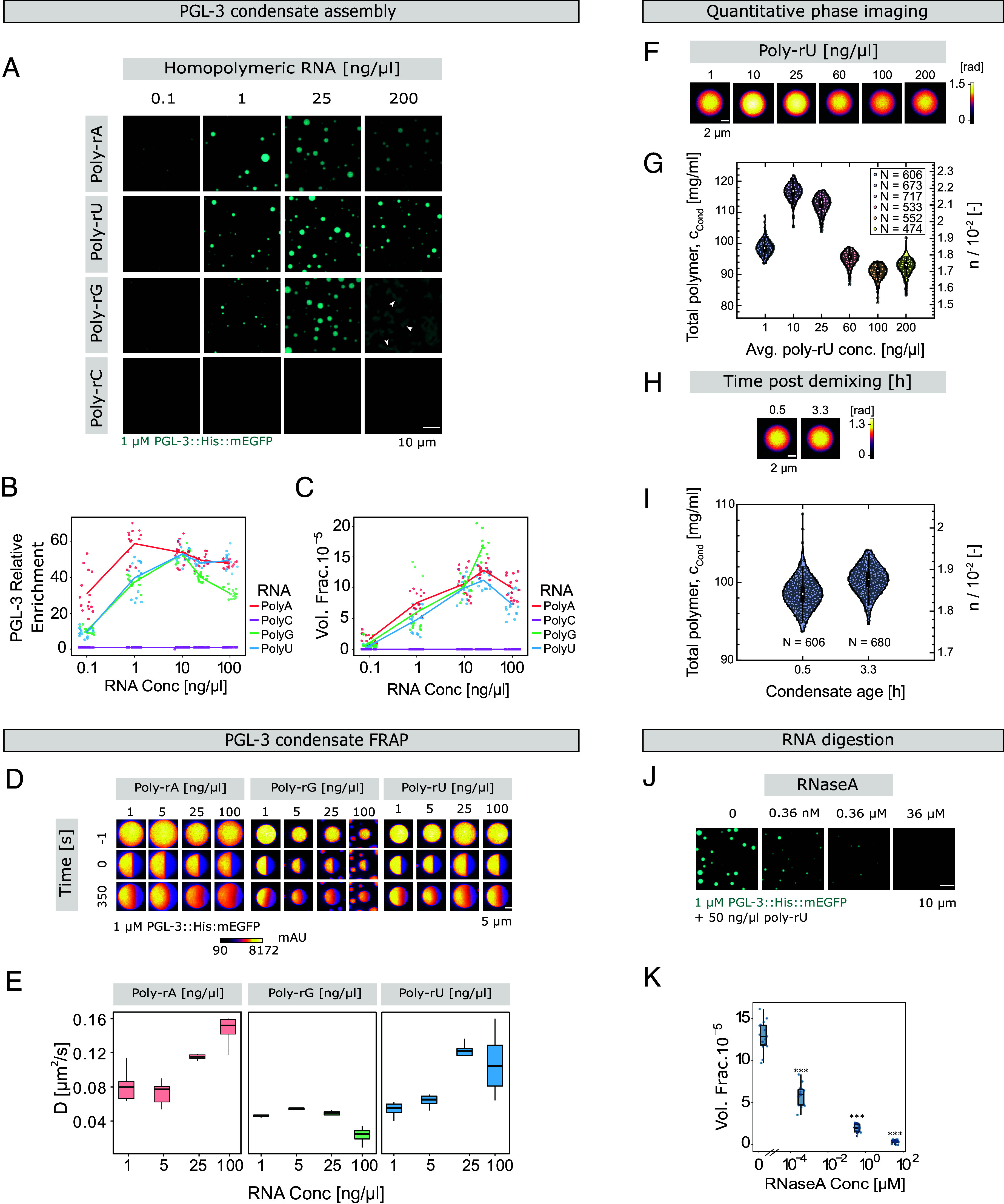
RNA sequence and concentration regulate the properties of PGL-3 condensates in vitro. (*A*) Maximum intensity projections of confocal z-slices of PGL-3::6xHis::mEGFP (1 μM) condensates with poly-rA, poly-rU, poly-rG, and poly-rC respectively (0.1 to 200 ng/μl RNA) were acquired 40 to 60 min after inducing condensate formation. Arrows point toward incompletely fused nonspherical condensates at 200 ng/μl of poly-rG (N = 3, n = 15). (*B*) The relative enrichment of PGL-3::6xHis::mEGFP within the droplet relative to the bulk increases with increasing RNA. Because of saturation inside the droplet, a further increase in RNA reduces the enrichment of PGL-3 inside the droplet relative to the bulk. All observations are represented with a line plot connecting the mean volume fraction and individual observations as scatter points. (*C*) The volume fraction of PGL-3::6xHis::mEGFP (1 μM) condensates shows an initial increase in RNA (except poly-rC), followed by a decrease. All observations are represented with a line plot connecting mean volume fraction and individual observations as scatter points. (*D*) Heat map of maximum intensity projections of confocal z-slices of PGL-3::6xHis::mEGFP (1 μM) condensates with either poly-rA, poly-rG, or poly-rU, and before (−1”), 0’’ or 350’’ after half-FRAP (N = 3, n = 9). (*E*) Diffusion constants of PGL-3::6xHis::mEGFP (1 μM) with poly-rA, poly-rG, and poly-rU titration respectively. Diffusion constants of PGL-3::6xHis::mEGFP increase for poly-rA, saturate for poly-rU after an initial increase, while it decreases for poly-rG. (*F*) Quantitative phase contrast images of PGL-3::6xHis::mEGFP (3 μM) condensates upon increasing poly-rU concentration. Colorbar gives local optical phase shift in radians. (*G*) Distributions of total polymer concentration (*Left*) and refractive index difference (*Right*) measured by QPI for condensates formed from PGL-3::6xHis::mEGFP (3 μM) and poly-rU (1 to 200 ng/μl). Colored circles correspond to individual measurements, white circles denote medians, thick black bars are the interquartile range, and whiskers extend 1.5× beyond the interquartile range. The total polymer concentration increases at first before decreasing subsequently again. (*H*) Quantitative phase contrast images of condensates formed from PGL-3::6xHis::mEGFP (3 μM) with poly-rU (1 ng/μl) at 0.5 and 3.3 h after inducing phase separation. (*I*) Distribution of total polymer concentration (*Left*) and refractive index difference (*Right*) measured by QPI for the conditions in (*H*). Box and whiskers are as in (*G*), and the 0.5 data are reproduced from (*G*) to ease comparison. The distribution mean increases by only 1.8% over approximately 3 h. (*J*) Maximum intensity projections of confocal z-slices of PGL-3::6xHis::mEGFP (1 μM) condensates with poly-rU (50 ng/μl) were acquired 60 min after adding RNaseA (0 to 36.5 μM) (N = 3, n = 15). (*K*) The volume fraction of PGL-3::6xHis::mEGFP (1 μM) condensates with poly-rU (50 ng/μl) decreases significantly with increasing RNaseA concentration. ***P < 0.001.

We next wanted to reconstitute RNA-dependent assembly of PGL condensates. This has previously proven to be challenging (19), because when PGL-3 was added in the presence of RNA, it hardens quickly and the dynamics of the proteins slow down (*SI Appendix*, *Supporting Information Text*). In order to address this, we investigated the role of RNA sequence and concentration in regulating the assembly and biophysical properties of PGL-3 condensates in vitro. The concentration of PGL-3 in embryos is around 0.5 μM (19). Similar to previous observations (19), we found that RNA is required to form condensates in vitro with 0.5-1 μM PGL-3. It has been hypothesized that mRNA in vivo is unwound by helicases which prevents its trapping in P granules (19, 22). To mitigate this effect by means of unfolded RNA, we tested the effect of four different homopolymeric RNA species, denoted poly-rC, poly-rG, poly-rA, and poly-rU, on the formation of condensates with 1 μM PGL-3. PGL-3 condensates did not assemble in the presence of poly-rC over a broad range of concentrations (Fig. 2*A*). PGL-3 incubated with poly-rG did form condensates, though these were nonspherical at the highest concentrations (Fig. 2*A*). PGL-3 condensates also formed with poly-rA and poly-rU and, unlike the poly-rG case, these were spherical over a broad range of concentrations (Fig. 2*A*). For the latter RNA types the PGL-3 enrichment inside condensates relative to the dilute phase increases with RNA concentration until 1 or 10 ng/µl, respectively, and subsequently stagnates or drops slightly at higher concentrations. We note that the enrichment decrease at the highest concentrations is largest for poly-rG (Fig. 2*B*). For poly-rA and poly-rU, the dense-phase volume fraction estimated from fluorescence microscopy is nonmonotonic, increasing to a maximum around 25 ng/uL RNA before decreasing at higher concentrations (Fig. 2*C*). While the dense phase volume fraction increased continuously until 25 ng/uL poly-rG as well, it is unclear whether the trend is nonmonotonic in this case because the irregularities in condensate shape precluded accurate volume assessment at higher RNA concentrations. FRAP analysis (Fig. 2 *D* and *E*) showed that PGL-3 diffusion coefficients at low poly-rU and poly-rA concentrations are similar to the average value of *D* = 0.056 μm^2^/s measured previously for PGL-3 in vivo (23). For instance, we measure diffusion coefficients of 0.04 to 0.08 μm^2^/s for PGL-3 inside condensates prepared with 1 to 5 ng/μl poly-rU in vitro.

Given that 91% of *C. elegans* mRNAs contain stretches of uridines in the 3’ UTR (24), we subsequently focused on condensates assembled from poly-rU. Quantification of the combined mass concentration of the biopolymers PGL-3 and poly-rU inside the condensates with Quantitative Phase Imaging (25) showed a nonmonotonic behavior: As the total poly-rU concentration is increased, the total polymer concentration inside condensates initially increases and then decreases beyond 10 ng/μl RNA (Fig. 2 *F* and *G* and *SI Appendix*, Fig. S1). Together with the diffusion (Fig. 2 *D* and *E*), enrichment (Fig. 2*B*), and volume fraction (Fig. 2*C*) measurements, these data reveal a complex dependence of condensate composition and physical properties on the average RNA concentration in this region of phase space.

Previous work has shown that reconstituted PGL-3 condensates display time-dependent properties, including increases in viscosity and density as the droplets age (19, 25, 26). Since reductions in molecular mobility associated with aging might impair the study of condensate disassembly, we next sought to assess time dependence of our reconstituted PGL-3/poly-rU condensates. First, we used quantitative phase imaging to look for changes in the total dense-phase polymer concentration over approximately 3 h (Fig. 2 *H* and *I*). We find that the mean RI difference increases from 0.018419 ± 0.000012 (SEM, N = 606 droplets) at t = 0.5 h to 0.018750 ± 0.000010 (SEM, N = 680 droplets) at t = 3.3 h, corresponding to an increase in the dense-phase polymer concentration from 98.55 ± 0.06 mg/ml to 100.32 ± 0.05 mg/ml. While this 1.8% increase in polymer density is indicative of aging, we note that the effect is small, particularly compared to the ~33% variation in density we observe as a function of RNA concentration at early times (Fig. 2*G*). We next compared the diffusion coefficient of PGL-3-mEGFP at 0.5 h (D_early_) and 3 h (D_late_) after phase separation of 1 μM PGL-3 with increasing amounts of poly-rU (*SI Appendix*, Fig. S2). In the absence of RNA, we find D_early_/D_late_ > 1, indicating diffusion slows down over time, which is consistent with previous reports of aging in PGL-3 condensates (25, 26). However, we also find that this diffusion coefficient ratio decreases monotonically as increasing concentrations of poly-rU are added to the system (*SI Appendix*, Fig. S2). Together, these data demonstrate that while RNA does not completely abolish aging or time-dependent diffusivity of PGL-3 in the dense phase, it does suppress the slow-down of diffusional dynamics often associated with condensate aging.

### MEX-5 Binding to Poly-rU Via the Zinc Finger Domain Is Necessary for the Disassembly of PGL-3/poly-rU Condensates In Vitro.

To corroborate our in vivo results showing that P granules disassemble after the addition of RNaseA (Fig. 1), we determined the relationship between the volume fraction of PGL-3/poly-rU condensates formed in vitro and RNaseA concentration. The volume fraction was calculated from the z-projected droplet radius. Fig. 2 *J* and *K*, shows that the volume fraction of condensates decreases with increasing RNaseA concentration. This suggests that RNA degradation post-PGL-3 condensate formation can induce PGL-3 condensate disassembly.

Previous work has shown that MEX-5 prevents the assembly of liquid drops of minimal P granule condensates by binding to RNA (19). This was based on data showing that a MEX-5 fragment containing the RNA-binding zinc finger could prevent drops of PGL-3 assembling in the presence of mRNA. However, because of PGL-3 droplet hardening in the presence of mRNA in vitro, it was not possible to study the role of MEX-5 in disassembling P granule protein droplets formed in vitro. To set up such an assay, we purified functional full-length MEX-5 (MEX-5) and its zinc finger deletion variant (MEX-5ΔZF) (*SI Appendix*, Fig. S3 and *Supporting Information Text*). Native gels show a predominant monomer fraction and a smaller fraction of dimer for both MEX-5 and MEX-5ΔZF (*SI Appendix*, Fig. S3). This supports the prediction of MEX-5 ability to self-associate plausibly by the glutamate-rich N terminus (16).

**Fig. 3.**
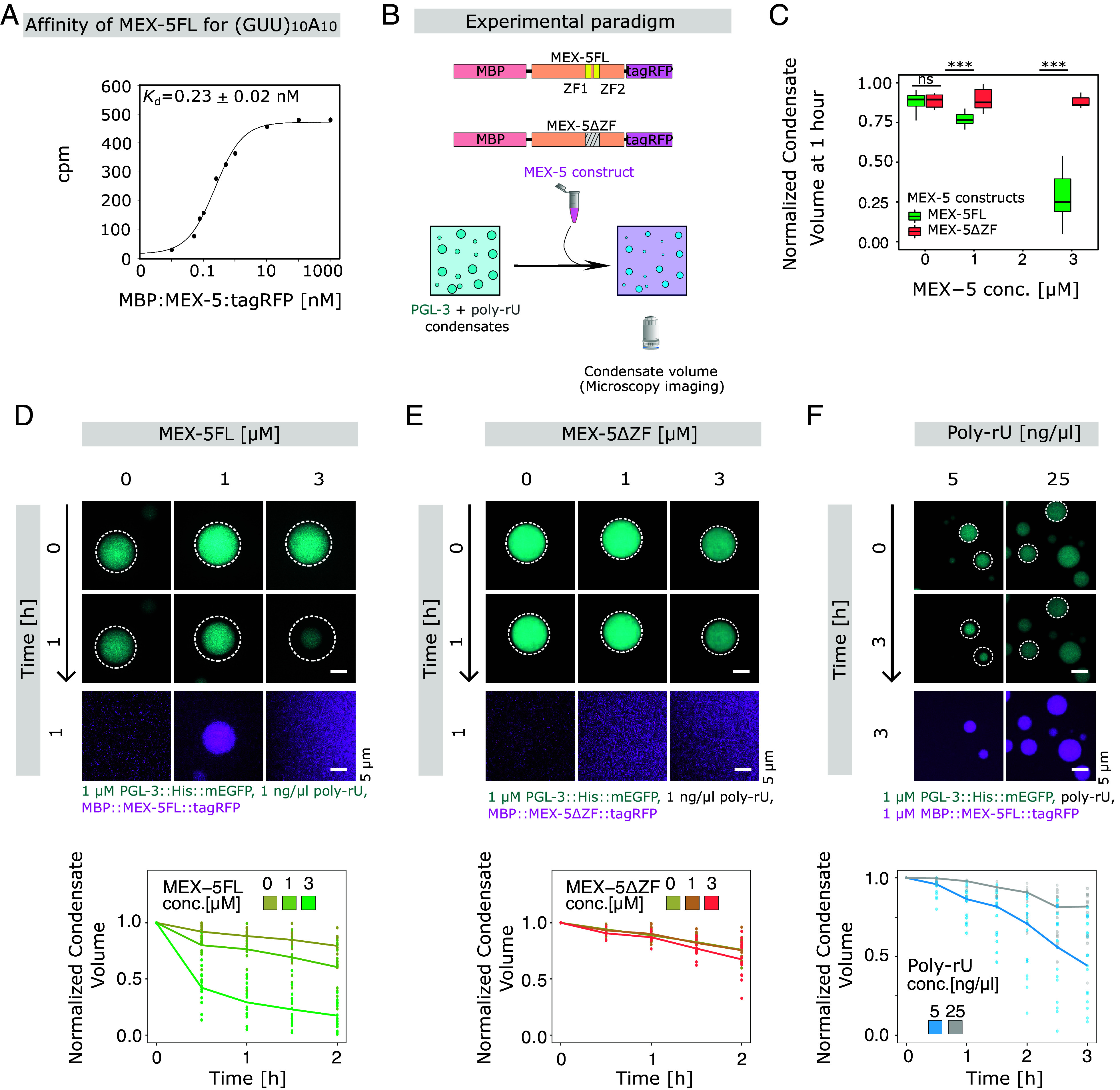
The MEX-5 zinc finger domain is necessary for the disassembly of PGL-3/poly-rU condensates in vitro. (*A*) Binding of MBP-MEX-5-tagRFP to RNA in vitro in filter binding assay at 100 mM KCl. The plot shows the amount of (GUU)_10_A_10_ RNA oligo bound MBP-MEX-5-tagRFP as a function of protein concentration. The solid curve corresponds to a fit of the form y = A + B/(1 + *K*_d_/x), where A and B are constants, and *K*_d_ is the dissociation constant of binding between MBP-MEX-5-tagRFP and radiolabeled RNA (cpm). (*B*) Illustration of the experimental paradigm with addition of either MEX-5 constructs; MBP-MEX-5-tagRFP and MBP-MEX-5ΔZF-tagRFP to the preassembled PGL-3/poly-rU condensates in vitro and assessment of disassembly by microscopy. (*C*) The normalized volume of PGL-3/poly-rU condensates 1 h after introducing MBP-MEX-5-tagRFP shows a significant decrease at both 1 and 3 μM compared to MBP-MEX-5ΔZF-tagRFP (N = 3, n = 15) (*D*) Maximum intensity projections of confocal z-slices of PGL-3-6xHis-mEGFP (1 μM) condensates with poly-rU (1 ng/μl) were acquired 0 min and 1 h after adding 0-3 μM of MBP-MEX-5-tagRFP and (*E*) MBP-MEX-5ΔZF-tagRFP. Condensate size appears to decrease only in MBP-MEX-5-tagRFP and not MBP-MEX-5ΔZF-tagRFP. The dotted circle marks the periphery of the condensate at 0 min. The presence of the ZF domain is necessary for the mild enrichment of MEX-5 inside condensates. The broad range of MBP-MEX-5-tagRFP enrichment at 3 μM is elaborated in *SI Appendix*, Fig. S4. The normalized volumes of condensate are represented with a line plot connecting the mean normalized volume and individual normalized volume observations as scatter points. (*F*) Maximum intensity projections of confocal z-slices of PGL-3-6xHis-mEGFP (1 μM) condensates at two different concentrations of poly-rU (5 and 25 ng/μl) were acquired 0 min and 3 h after adding MBP-MEX-5-tagRFP at 1 μM. Condensate size appears to decrease more at lower poly-rU (5 ng/μl) compared to a higher concentration (25 ng/μl). MBP-MEX-5-tagRFP appears to partition similarly into condensates with different poly-rU concentrations (N = 3, n = 15). The normalized volume of condensate shows a steeper decrease in volume at lower poly-rU (5 ng/μl) compared to a higher concentration (25 ng/μl) in the presence of fixed MBP-MEX-5-tagRFP concentration. All observations are represented with a line plot connecting the mean normalized volume and individual normalized volume observations as scatter points. ***P < 0.001.

In order to compare the interaction of the here-employed full-length MEX-5 with (GUU)_10_A_10_ RNA which was used previously with the MEX-5ZF fragment (19), the affinity to this RNA type was measured using a filter binding assay. We find that full-length MEX-5 binds with a 50-fold higher affinity to (GUU)_10_A_10_ RNA compared to the previously reported values for the MEX-5ZF fragment (Fig. 3*A*). Further, we assessed the effect of MEX-5 on the dynamics of the liquid PGL-3/poly-rU condensates (hereafter referred to as PGL-3/RNA condensates) (Fig. 3*B*). While MEX-5 is able to shrink preassembled PGL-3/RNA condensates quantitatively over the span of an hour, the zinc finger deletion mutant MEX-5ΔZF does not change the condensate volumes (Fig. 3*C*). The addition of MEX-5 to preassembled PGL-3/RNA condensates resulted in a MEX-5 concentration-dependent progressive decrease in their volume over a 2-h window (Fig. 3*D*). A similar decrease in the condensate volume was not observed upon addition of MEX-5ΔZF in different concentrations (Fig. 3*E*). The high affinity of full-length MEX-5 for RNA sequences and faster disassembly kinetics of condensates with lower poly-rU concentration (Fig. 3*F*) supports a previously proposed RNA binding competition model in which MEX-5 dissolves PGL-3/RNA condensates by sequestering RNA (12, 19).

### MEX-5 Shifts the Phase Separation Boundary in PGL-3/poly-rU Condensates In Vitro.

**Fig. 4.**
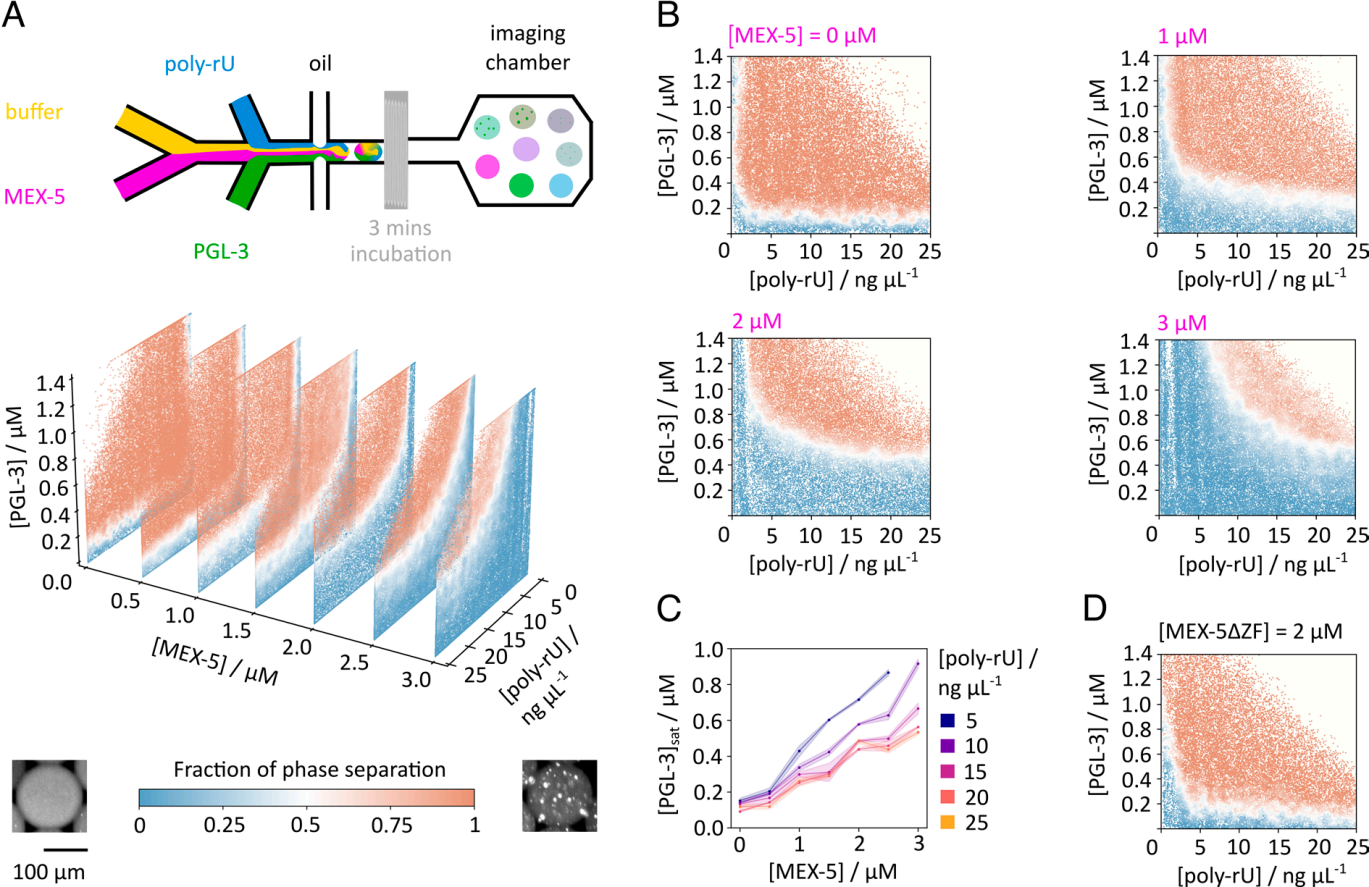
MEX-5 shifts the phase separation boundary in a PGL-3/poly-rU phase diagram. (*A*) 3D phase diagram stack of seven 2D slices of PGL-3, poly-rU, and MEX-5 in 200 mM KCl was recorded at 22 °C. The component concentrations in microdroplet were varied through alteration of the flow rates on chip. After an incubation time, the droplets were imaged. The probability of phase separation from image classification is depicted on a scale from phase separated (red) to homogeneous (blue), revealing a sharp phase boundary. The color bar applies to the diagrams in (*A*), (*B*), and (*D*). (*B*) 2D visualizations of selected slices of the stack in (*A*). MEX-5 leads to a gradual boundary shift. (*C*) Dose response of the PGL-3 saturation concentration against MEX-5 concentration at different poly-rU concentrations. At higher RNA concentrations, the phase separation inhibition becomes less pronounced and levels off between 20 ng/μL (22 nM) and 25 ng/μL (28 nM). SD of the phase separation probabilities between 0.46 and 0.54 at the respective RNA concentrations ±0.2 are depicted as transparent error bands. (*D*) The MEX-5 zinc-finger deletion mutant shows almost no effect on the PGL-3/poly-rU phase boundary for low poly-rU and PGL-3 concentrations.

Taken together, our data so far show that it is possible to assess the activity of MEX-5 in controlling the stability of a simplified minimal P granule system using PGL-3 and a single RNA species. In order to investigate the effect of MEX-5 on PGL-3/poly-rU-dependent phase separation, we employed a combinatorial droplet microfluidic platform (27–29). In this method, a large number of individual water-in-oil droplets are prepared on chip, each composed of a different concentration of solutes, as controlled through variation of the component flow rates (Fig. 4*A*). Postincubation, the aqueous droplets are subsequently imaged and classified for phase separation by means of a convolutional neural network to generate a phase diagram.

We first recorded a 3D phase diagram by varying the concentrations of MEX-5, poly-rU, and PGL-3 (Fig. 4*A*). The color coding of phase separated (red) and mixed (blue) corresponds to detected condensates in the PGL-3 channel. We find that increasing MEX-5 concentration leads to both higher PGL-3 and higher poly-rU concentrations necessary to induce condensate formation, confirming our previous observations in the time-dependent assay. Interestingly, addition of MEX-5 yields a shift in the phase boundary toward increasing both the saturation concentration of PGL-3 and poly-rU (Fig. 4*B*).

We further find that at low RNA concentrations, MEX-5 has the highest effect on the phase boundary which becomes less pronounced for higher poly-rU concentrations and levels off above 20 ng/μL. Here, the lower end of the binodal is reached and the phase boundary shift becomes independent of the RNA concentration (Fig. 4*C*). We conclude that the occupation of RNA binding sites at low concentrations becomes more challenging as MEX-5 competes stoichiometrically for them with PGL-3.

To confirm that MEX-5 affects the phase boundary due to its ability to bind to poly-rU as hypothesized above, the zinc finger deletion mutant MEX-5∆ZF was added to the minimal P granule system (Fig. 4*D*). Compared to the boundary of the PGL-3/poly-rU phase diagram without addition of MEX-5 there is only a very slight change visible at low RNA concentrations below 4 ng μL^−1^ (4.4 nM) which lies within the error of the measurement. Thus, the resulting phase diagram confirms that the boundary shift induced by MEX-5 is correlated with its RNA binding activity.

### Mechanism of Action of MEX-5.

**Fig. 5.**
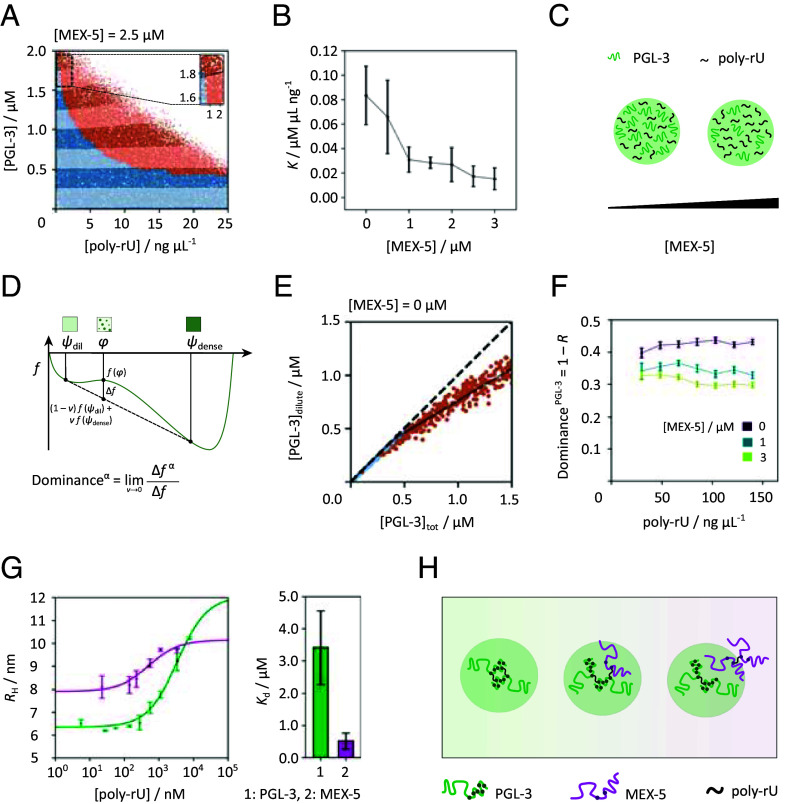
Thermodynamic parameters of RNA association with MEX-5 and hypothesized mechanism. (*A*) Exemplary phase diagram at [MEX-5] = 2.5 μM with dilute phase bands depicted at assigned protein concentrations. The positive slope of the dilute phase contours provides information about PGL-3 and RNA partitioning into the dense phase. (*B*) Measuring the dilute phase contour slopes *K* between 1.55 and 1.95 μM PGL-3 at the phase boundaries shows a decreasing *K* upon addition of MEX-5. Error bars correspond to the SD within the investigated window. (*C*) This suggests a lower PGL-3 to RNA ratio inside formed condensates upon MEX-5 concentration increase. (*D*) Maxwell construction showing the free energy before (*f* (ϕ)) and after ((1 − *ν*)*f* (*ψ*_dil_) + *νf* (*ψ*_dense_)) phase separation and a 1D representation (ϕ: total concentration of solutes in a sample containing α = 1,2, …N species, *ψ*: solute concentrations on the dilute and dense surfaces, *ν*: volume of the dense phase relative to the whole system) (33). (*E*) The Dominance can be obtained from the PGL-3 dilute phase response gradient as exemplarily shown for a slice at 30 ng μL^−1^ (33 nM). (*F*) The PGL-3 dominance decreases upon addition of MEX-5 leading to the assumption that MEX-5 directly interferes with PGL-3 in the phase separation mechanism. Error bars correspond to the SD of the fitted *R* values. (*G*) By means of microfluidic diffusional sizing, the hydrodynamic radii of MEX-5 (1 μM) and PGL-3 (1 μM) upon binding to poly-rU can be measured. The infliction points of the binding curves (black) correspond to the *K*_d_ which shows a sevenfold higher affinity to RNA by MEX-5 than PGL-3. Error bars correspond to the SD of the determined *K*_d_ values. (*H*) Proposed mechanism of RNA recruitment by MEX-5. Upon dissolving condensates, MEX-5 inhibits PGL-3/RNA interactions by forming MEX-5−RNA−poly-rU assemblies.

To further study the mechanism of action of MEX-5 on PGL-3/RNA condensates, we quantified the PGL-3 dilute phase concentration in each individual microfluidic droplet environment and studied its response when total concentrations of RNA, PGL-3, and MEX-5 were varied. Crucially, the dilute phase concentration change in PGL-3 is informative of solute partitioning (30, 31) and thermodynamics (32, 33) of phase separation, as it describes the thermodynamic demixing process. Briefly, in a PGL-3/RNA phase diagram, contours of constant dilute phase PGL-3 concentration can be evaluated from the data. In an exact two-component system, lines of constant dilute phase concentration in the phase-separated region coincide with tie lines connecting the dilute and dense phase compositions. In general, however, the presence of other solute species means the slope of these contours, denoted hereafter by *K*, provides a lower bound of the PGL-3/RNA tie line component ratio (32). The difference between *K* and the true tie line component ratio depends on the shape of the phase boundary, and this difference is the smallest in the region where the phase boundary is parallel to the PGL-3 axis. We visualize PGL-3 dilute phase contours by first defining threshold concentrations spaced by 0.25 μM along the PGL-3 axis, leading to 8 dilute phase bands, and then assign alternating light/dark shades of blue (mixed) and red (demixed) colors for data points in these bands (Fig. 5*A*), The boundary between light- and dark-shaded bands is continuous across the binodal and represents a “PGL-3 dilute-phase contour”. In the homogeneous region, the contours are parallel to the RNA axis as expected, since the dilute phase concentration is equal to the total PGL-3 concentration. In the phase-separated region, the contours exhibit a positive slope, indicating PGL-3 and RNA copartition into the condensates. To estimate the PGL-3/RNA tie line component ratio, we observe that the phase boundary between 1.55 and 1.95 μM PGL-3 is mostly parallel to the PGL-3 axis, and as such we compute *K* in this window. By carrying out this procedure at different MEX-5 concentrations, we show that *K* decreases for increasing MEX-5 (Fig. 5*B*). This suggests that the stoichiometry changes toward a lower PGL-3 to RNA ratio inside the dense phase (Fig. 5*C*), revealing that introduction of MEX-5 can also affect the stoichiometry and composition of PGL-3/RNA condensates.

Measurement of the protein dilute phase concentration further allows us to extract information on the free energy contributions that drive phase separation (31–33). Specifically, we determine the free energy contribution from PGL-3 relative to the total free energy change upon phase separation, and this fraction is hereon referred to as Dominance (Fig. 5*D*). Experimentally, it could be obtained by plotting the PGL-3 dilute phase concentration against its total concentration (Fig. 5*E*) at a fixed poly-rU concentration. Provided that the condensate volume is small, the dominance is given by Dominance = 1 − *R* where *R* is the response gradient of the dilute phase which corresponds to the slope of a linear regression through the points in the phase-separated regime in the graph (Fig. 5*E*). The PGL-3 dominance decreases for increasing MEX-5 concentrations between 30 (33) and 140 ng μL^−1^ (156 nM) poly-rU (Fig. 5*F*). Hence, the introduction of MEX-5 decreases the relative contribution of PGL-3 to the free energy change driving phase separation. Combined with the condensate-dissolving property of MEX-5, we deduce that MEX-5, as a modulator, weakens interactions involving PGL-3 (32). Since MEX-5 is binding RNA, a possible mechanism of dissolution is MEX-5 competing with PGL-3 for RNA binding sites, thus implicitly disrupting PGL-3/RNA binding and further corroborating that MEX-5 influences the collective interactions of PGL-3 with other components in the condensate environment.

We also used a microfluidics system to provide an orthogonal measurement for the affinity of MEX-5 for RNA. In this method, the size (hydrodynamic radius) of the proteins is determined by measuring their diffusive motion on chip (34). The affinities measured for PGL-3 and MEX-5 in solution with diffusional sizing are lower than for the filter binding assays (Fig. 3*A*), but show the same trend. MEX-5 has a sevenfold higher binding affinity to poly-rU than PGL-3. MEX-5∆ZF does not show binding to poly-rU (*SI Appendix*, Fig. S5). The measured *K*_d_ for MEX-5 is 0.52 μM while PGL-3 has a *K*_d_ of 3.42 μM poly-rU.

## Discussion

Regulation of the formation and dissolution of biomolecular condensates is a central problem in cell biology. In this paper, we demonstrate that condensates formed in a test tube from the *C. elegans* P granule protein, PGL-3, and RNA, can be dissolved by addition of MEX-5 protein.

Our work is based on a previous in vitro assay (19) in which condensate formation from PGL-3 and RNA could be prevented by the RNA binding fragment of the protein MEX-5. An RNA competition mechanism was proposed, by which MEX-5 prevents P granule assembly at the anterior of the *C. elegans* embryo by sequestering RNA. This previous assay was however unable to test the role of MEX-5 in dissolving minimal P granule condensates because the PGL-3/RNA condensates hardened quickly (19). Identifying synthetic RNA sequences that maintain RNA/PGL-3 condensate liquidity, and succeeding in making full-length MEX-5, which was not possible in previous P granule reconstitution assays (12, 19), allowed the development of an assay that could be used to study MEX-5 regulation of minimal P granule condensates. Application of poly-rU yielded important biophysical similarities with PGL-3 condensates in vivo, as in vitro PGL-3 condensates with poly-rU exhibit diffusion coefficients comparable to in vivo PGL-3 (23).

Reconciling the phase boundary shifts, dilute phase contours, and relative free energy contributions upon addition of MEX-5, we can propose a refined minimal P granule condensate dissolution mechanism: Condensates form largely based on interactions of PGL-3 with RNA (Fig. 5 *H*, *Left*). Assuming that within the PGL-3/RNA condensate RNA molecules are typically bound by multiple PGL-3 molecules at the same time and PGL-3 itself is able to undergo interactions with multiple RNAs, then upon addition of MEX-5, PGL-3−RNA−MEX-5 assemblies can form (Fig. 5 *H*, *Middle*). However, binding of MEX-5 to the RNA in this way will block RNA binding sites for the interaction with PGL-3, therefore, affecting the RNA/PGL-3 interactions and decreasing the free energy contribution of PGL-3 to condensate formation. This “indirect” binding of MEX-5 to PGL-3 through RNA helps rationalize the shift on both RNA and PGL-3 axes in the phase diagram. Furthermore, the decrease in the PGL-3 to RNA ratio is consistent with MEX-5 entering the dense phase of the formed condensates. Finally, MEX-5 sequesters RNA completely (Fig. 5 *H*, *Right*) which leads to full PGL-3/RNA condensate dissolution. Taken together, MEX-5 does not solely act by RNA binding competition but likely interferes directly with the PGL-3−poly-rU interaction driving the phase transition.

The in vitro RNA binding measurements performed here have confirmed earlier observations that MEX-5 has a significantly higher binding affinity to RNA compared to PGL-3 (24). However, previously reported measurements using a filter binding assay displayed a 20-fold higher affinity of the MEX-5 zinc finger domain to (GUU)_10_A_10_ mRNA and overall lower *K*_d_ values (19) than determined here. While in filter binding assays complexes are attached to a membrane and nonbound compounds are removed in filtration, microfluidic diffusional sizing measures the hydrodynamic radii of species in solution. Deviations in binding affinities might be a result of different salt concentrations in the respective experimental setups and may also be related to differences in the interaction geometry as surface-based interactions fail to accurately represent binding events under in-solution conditions (35). Another factor is the different type of employed RNA and the fact that poly-rU is provided in a range of different lengths, which might lead to differences in dissociation constants. The fact that excess MEX-5 is needed to dissolve PGL-3/RNA condensates despite its higher binding affinity could be because of the difference in binding sites. PGL-3 has 6 RNA-binding sites while MEX-5 has only 2 (19).

Both MBP::MEX-5::tagRFP and PGL-3::His::mEGFP were purified and used in their tagged form. It is anticipated that the fluorescent protein tags will alter the condensation behavior (36, 37). Indeed, in vitro, the saturation concentration decreases when the tagged PGL-3 concentration exceeds 20% (38). Our goal was to develop an in vitro system that is similar to the situation in *C. elegans* embryos in which MEX-5 or PGL-3 are fully tagged at the endogenous locus. Both PGL-3 and MEX-5 tagged CRISPR lines have brood sizes and fertility comparable to wild-type worms (*SI Appendix*, Fig. S6). In our in vitro assays MEX-5::tagRFP carried an additional N-terminal MBP tag to avoid its aggregation. Although tagged MEX-5::mEGFP seems fully functional in vivo, we were not able to construct a MEX-5::tagRFP line. Whether tagRFP requires a specific linker when fused to MEX-5 or whether other red fluorescent proteins like mScarlet are an option is currently under investigation. Given this, future work may prefer MEX-5::mEGFP variants. Although we see disassembly of condensates with the MEX-5 full-length but not with the MEX-5∆ZF variant missing the zinc finger, we can not exclude that the tags impair activity.

Our assay is a three-solute system involving RNA, MEX-5, and PGL-3, but it still differs significantly from the situation in the embryo. MEX-5 is a substrate for two kinases MBK-2 and PLK-1 which are activated during the Oocyte-to-Embryo transition (39–41). The uniform distribution and expansion of P granules into the anterior half of the one-cell embryo in *mbk-2* and *plk-1* mutants respectively, despite the maintenance of the anterior enrichment of MEX-5 by PAR-1 supports the interplay of multiple kinases in the spatiotemporal regulation of the disassembly of P granules (41–43). Future work will be required to study the effect of kinases on MEX-5 activity in vitro. In addition to the PGL proteins, MEX-5 regulates the gradient of the P granule protein MEG-3, an mRNA recruiting protein expressed only in early embryos (12) and required for P granule segregation. Analysis of transcripts bound to PGL-1 and MEG-3 revealed that MEG-3 is the major mRNA-recruiting protein, while PGLs bind to a very small subset of mRNA in embryos (44). It has been suggested that the MEG-3/4 are localized to the posterior end of the embryo by the MEX-5/6 gradient while in turn PGL-1/3 are localized to these granules and manipulate their size (11, 12). Therefore, it is likely that MEG proteins would have a significant influence on MEX-5-dependent PGL-3/RNA dissolution. Due to the use of homopolymeric RNA in our assays, we do not anticipate secondary structures except for G-quadruplexes. In vivo however, the presence of helicases like GLHs becomes crucial as they actively untangle secondary RNA structures (22). The role of changing RNA concentrations, sequence, and structure of RNA inside P granules in regulating the rate of disassembly in vivo is an exciting unexplored territory.

## Materials and Methods

### Protein Expression and Purification.

MBP::MEX-5::tagRFP and MBP::MEX-5ΔZF::tagRFP were purified from Tni insect cells using the baculovirus infection system (45). Insect Tni cells were harvested ~72 h after viral infection and lysed by means of an LM-20 microfluidizer (Microfluidics) in lysis buffer (25 mM HEPES pH 7.25, 300 mM arginine, 100 mM KCl, 20 μL Benzonase, 1 tablet of protease inhibitor (Thermo Scientific), 1 mM MgCl_2_, 2 tablets PhosStop (Roche), and 1 mM DTT for 100 ml lysis buffer). The lysates were centrifuged in a JA25.50 (Beckman-Coulter) rotor at 16,000 rpm for 30 min at 10 °C. The supernatant was incubated with amylose resin (NEB) washed in MBP binding buffer containing 25 mM HEPES pH 7.25, 300 mM arginine, 100 mM KCl, and 1 mM DTT on the rotor for 2 h at 4 °C. The beads were centrifuged at 800 rpm for 3 mins. The pelleted beads were then washed with MBP binding buffer at 800 rpm for 3 mins. This washing step was repeated 3 times. MEX-5 protein was eluted with MBP elution buffer containing 25 mM HEPES pH 7.25, 300 mM arginine, 100 mM KCl, 10 mM maltose, and 1 mM DTT. The elute from amylose beads was diluted 5.6× with Heparin dilution buffer containing 25 mM Tris pH 8, 1% glycerol, and 1 mM DTT to reach 50 mM KCl for binding to the heparin Sepharose beads. Equilibrated Heparin Sepharose beads and MBP purified MEX-5 in Heparin dilution buffer were incubated at room temperature for 3 min. MEX-5 was washed and eluted in heparin elution buffer at 25 mM Tris pH 8, 150 mM KCl, 1% glycerol, and 1 mM DTT.

PGL-3::6xHis::mEGFP was purified as before (19). All the insect cells used were infected with a baculovirus system (45). All the purified proteins were distributed into small aliquots, flash-frozen in liquid nitrogen, and stored at −80 °C (Table 1).

**Table 1.** Recombinant DNA used in the study

Recombinant DNA	Source
pOEM1-based plasmid for baculovirus expression of PGL-3-His6-mEGFP	Published (19)
pOEM1-based plasmid for baculovirus expression of MBP-PS-MEX-5-opt-TEVtagRFP	This Study (TH1767)
pOEM1-based plasmid for baculovirus expression of MBP-PS-MEX-5ΔZF-opt-TEVtagRFP	This Study (TH2549)

### RNaseA Injection in Worm.

Microinjection of worms as shown in Fig. 1 was performed in halocarbon oil on agarose pads. Young adult worms were picked and transferred into the oil. Worms were attached to the pad by pressing the head and tail firmly onto the pad using a worm pick (46). The diameter of the microcapillary needle containing RNaseA (Merck) or RNaseA (Merck) and RNaseOUT (Invitrogen) was injected (Eppendorf FemtoJet) into the germline syncytium. The worms were immediately transferred to clean agarose pads and washed with M9 buffer. The worms were mounted on an agarose pad using the method described in ref. 46 and imaged immediately every 15 min for 90 min using a Visitron spinning disc system with 63x/1.3 NA glycerol objective. The worm lines are tabulated below (Table 2).

**Table 2.** List of worm strains used in this study

Strain number	Genotype
TH561	PGL-3::mEGFP [pgl-3(dd29)(pgl-3::mEGFP)]
TH586	PGL-1::mEGFP [pgl-1(dd54)(pgl-1::mEGFP)]
TH626	MEX-5::mEGFP [mex-5(dd58)(mex-5::mEGFP)]; PGL-3::mCherry(knu99)

### RNA Homopolymer Titration Assay.

PGL-3-6xHis-mEGFP drops as shown in Figs. 2 and 3 were assembled by diluting the protein from a high salt-containing storage buffer (300 mM KCl) to a physiological buffer (150 mM KCl) by adding blank buffer containing homopolymeric RNA (0 mM KCl). Homopolymeric RNA used was poly-rA (Sigma-Aldrich, P9403), poly-rU (Sigma-Aldrich, P9582), poly-rG (Sigma-Aldrich, P4404), and poly-rC (Sigma-Aldrich, P4903). Condensates of PGL-3 were imaged within 40 to 60 min following PGL-3/RNA condensates assembly. Imaging specifications are below in *Materials and Methods*. All in vitro assays with PGL-3 and RNA were carried out in a physiological buffer (25 mM HEPES pH 7.25, 150 mM KCl, and 1 mM DTT).

### FRAP.

A linear intensity profile across the half-FRAP ROI as shown in Fig. 2 was obtained by selecting a six-pixel wide stripe in FIJI (47) at the maximum diameter of the droplet, covering the dark and bright halves, averaging along the short dimension and subtracting camera background. The droplet edges are found automatically using the intensity drop-off at the edges prior to bleaching. This procedure is carried out for every time frame of the recovery. Subsequently, the first profile is used as the initial condition for a 1D diffusion equation with experimentally determined boundary conditions (48). Fitting also follows as described in ref. 48. The entire code can be found at https://git.mpi-cbg.de/hubatsch/droplet-frap/.

### Dilute Phase Measurement of PGL-3 and RNA.

Dilute phase measurement of PGL-3 was done using QubitTM Protein Assay Kits (Thermo Fisher Scientific) on the Qubit Fluorometer (Thermo Fisher Scientific). RNA measurements were done using NanoPhotometer (IMPLEN).

### Quantitative Phase Imaging and Analysis.

Quantitative phase imaging as shown in Fig. 2 of multicomponent condensates was undertaken as previously described (25) using a coherence-controlled holographic microscope (Q-Phase G2, Telight, Brno, CZ) based on (49).

Samples containing RNA/PGL-3 condensates were prepared as described above. However, to increase the accuracy of the phase imaging measurements, we increased the average droplet size by preparing samples at a higher average PGL-3 concentration of 3 μM. Immediately after preparation, 5 μL of sample was loaded into a temperature-controlled flow cell. The flow cell was prepared from PEGylated coverslips (30 × 24 × 0.17 mm^3^) adhered by heat to a sapphire slide (75 × 25 × 1 mm^3^) with parafilm strips. The ends of the channel were sealed with two-component silicone glue Twinsil (Picodent, Wipperfürth, DE), and the droplets were allowed to settle for ~10 min prior to measurement. The temperature for the slide was maintained at 22 °C for all measurements using water-cooled Peltier elements as previously described (50).

Samples were illuminated with an LED light source filtered at λ = 650 nm by a 10-nm bandwidth notch filter through a condenser set to an NA of 0.30. Images were collected with a 40× dry objective (0.9 NA, Nikon). Typically, hologram *z*-stacks (*dz* = 0.2 µm) were acquired for several fields of view with the first plane taken close to the surface of the cover glass. SophiQ software (Telight, Brno, CZ) was used to construct amplitude and compensated phase images from the raw holograms.

All phase images were subsequently analyzed using custom code written in MATLAB, as described previously (25). Briefly, individual droplets were identified in each image by intensity-based segmentation. For each identified droplet, the refractive index difference, Δn, and the geometric parameters $R,x_{c},y_{c}$, and $Z_{\mathrm{eq}}$ denoting droplet radius, (x, y)-coordinates of centroid, and height of the droplet’s equatorial plane above the coverslip, respectively, were determined by fitting the measured phase shift Δφ within a region of interest centered on the object to,

[1] $$\Delta \varphi \left(x,y\right)=\frac{2\pi }{\lambda }\Delta nH_{\mathrm{cap}}\left(x,y|R,x_{c},y_{c},Z_{\mathrm{eq}}\right)+\varphi_{0}+A\left(Z_{\mathrm{eq}},R\right).$$

Here, $H_{\mathrm{cap}}$ is the traversed height of a spherical cap, $\varphi_{0}$ is a constant phase offset and $A\left(Z_{\mathrm{eq}},R\right)$ is a regularization function [please see (25) for details]. Track.m (https://site.physics.georgetown.edu/matlab/index.html) was used subsequently to track all droplets within a z-stack through z. For each droplet, representative parameters are taken as those for which the Adj. R^2^ from the fit was largest. All detected objects for which the best fit had Adj. R^2^ < 0.98 were discarded. In each case, we found the ∆n of individual droplets to be tightly and symmetrically distributed around a central mean.

### Condensate Composition: Estimation and Measurement.

In this work, we use two different analytic approaches to infer the composition of poly-rU PGL-3/RNA condensates from the refractive index difference extracted from quantitative phase imaging. The first represents an estimate of the local polymer mass concentration in the PGL-3/poly-rU condensate is employed in Fig. 2*G*. Below, we introduce the basic optical model and the two analytic approaches in turn.

Following previous work (25), we employ a simple linear model for the refractive index difference between the PGL-3/poly-rU condensate and the surrounding dilute phase

[2] $$\Delta n=\frac{\mathrm{dn}}{{\mathrm{dc}}_{p}}\left(c_{p}^{\mathrm{cond}}-c_{p}^{\mathrm{dil}}\right)+\frac{\mathrm{dn}}{{\mathrm{dc}}_{r}}\left(c_{r}^{\mathrm{cond}}-c_{r}^{\mathrm{dil}}\right),$$

where $c_{i}$ and $dn/dc_{i}$ represent the concentration and refractive index increment of species *i*, respectively. The subscripts *p* and *r* denote protein and RNA, respectively, while the superscripts specify condensed- and dilute-phase concentrations, respectively. This model states that the refractive index difference arises from differences in the concentrations of protein and RNA in the two phases. The refractive index increments indicate, for each polymer species, how much a given concentration imbalance contributes to the refractive index difference. For $dn/dc_{r}$, we use the 0.1665 ± 0.0046 ml/g value measured previously for poly-rA RNA (25). Using a calculator tool described in ref. 51, we estimate $dn/dc_{p}$ from the amino acid sequence of PGL-3 to be 0.1869 ± 0.0051 ml/g. Note that the quoted uncertainty in $dn/dc_{p}$ is required for subsequent error propagation and was estimated from the relative uncertainty of the $dn/dc_{r}$ measurement as $\delta \left(dn/dc_{p}\right)\approx \delta \left(dn/dc_{r}\right)\times \left[\left(dn/dc_{p}\right)/\left(dn/dc_{r}\right)\right]$.

We next sought to obtain from the model in Eq. (**2**) an estimate of the overall polymer mass density in the PGL-3/poly-rU condensates. For this, we make two reasonable assumptions that enable simplifying approximations. The first assumption is that the concentrations of the protein and RNA in the dilute phase are both so low as to be negligible in comparison to the condensed-phase concentrations. With this assumption, we approximate the dilute-phase concentrations in Eq. (**2**) as zero. Given our subsequent finding that condensed-phase concentrations are 100- to 6,000-fold higher than the dilute-phase concentrations in the region we explored, this assumption introduces only a small overestimation in the total polymer mass concentration of at most 1%. The second assumption is that the refractive index increments for PGL-3 and poly-rU are very similar. Indeed, our estimates above place them within 11% of each other. With this assumption, we approximate $dn/dc_{r}\approx dn/dc_{p}$ in Eq. (**2**). With these approximations in place, the model simplifies to

[3] $$\Delta n≅\frac{\mathrm{dn}}{{\mathrm{dc}}_{p}}\left(c_{p}^{\mathrm{cond}}+c_{r}^{\mathrm{cond}}\right)=\frac{\mathrm{dn}}{{\mathrm{dc}}_{p}}c_{\mathrm{polymer}}^{\mathrm{cond}}.$$

The distribution of total polymer mass concentrations presented in Fig. 2 *G* and *H* were calculated from Eq. (**3**), using the refractive index difference distribution measured for individual PGL-3/poly-rU condensates by quantitative phase imaging and $dn/dc_{p}$ = 0.1869 ml/g.

### Postassembly of PGL-3/poly-rU Condensate RNaseA Addition Assay.

PGL-3-6xHis-mEGFP (25 mM HEPES at pH 7.25, 1 mM DTT, 300 mM KCl) was induced to assemble by lowering salt concentration by adding dilution buffer containing poly-rU with no KCl to give a final buffer composition of 25 mM HEPES at pH 7.25, 1 mM DTT, and 150 mM KCl. RNaseA (Merck) was added 40 to 60 min after poly-rU/PGL-3 condensate formation and imaged after 1 h incubation. Imaging specifications are below in *Materials and Methods*, Image acquisition.

### Filter Binding Assay to Test Binding Between MEX-5 and RNA.

Protocol published in ref. 19 was used.

### In Vitro Assays: PGL-3/RNA Condensate Disassembly Assay.

PGL-3 drops as shown in Fig. 3 were assembled by diluting the protein from a high salt-containing storage buffer (300 mM KCl) to a physiological buffer (150 mM KCl) by adding blank buffer containing poly-rU (0 mM KCl). The PGL-3/RNA condensates were centrifuged (Eppendorf) using at 100 g with acceleration setting at 9 and deceleration setting at 9 for 20 to 25 min at room temperature immediately after inducing phase separation. All in vitro assays with PGL-3 and RNA were carried out in physiological buffer (25 mM HEPES pH 7.25, 150 mM KCl, 1 mM DTT) at a volume of 20 μl. 2 μl of MEX-5 constructs (30, 10, 0 μM) in 25 mM Tris pH 8, 150 mM KCl, and 1 mM DTT was added to the PGL-3/RNA condensate assay. Images were acquired using an inverted Andor STORM Spinning disc microscope immediately after MEX-5 addition every 30 min for 2 h.

### Fluorescence Imaging.

The confocal z-slices of PGL-3/RNA condensates shown in Fig. 3 were imaged with an inverted Andor STORM Spinning disc Microscope, Andor iXON 897 EMCCD camera, 60x/1.2 Plan Apochromat VC, Water, NIKON Objective. At 10 to 11 μm z depth with 0.7 μm z step.

### Segmentation of PGL-3 Condensates In Vitro.

In vitro, PGL-3/RNA condensates from Fig. 3 were segmented using a custom-written Fiji Macro pipeline. In brief, image stacks were analyzed as two-dimensional (2D) maximum projections. All image stacks were first subtracted by the dark intensity count of the camera. Images are subjected to filtering. The mask was obtained from the segmentation of the PGL-3/RNA condensates. To measure the volume fraction of PGL-3/RNA condensate and partition coefficient of protein maximum intensity projections, segmented PGL-3/RNA condensates were then divided into PGL-3/RNA condensate (in) values and bulk (out) values. Radii of segmented PGL-3/RNA condensates were measured to compute the volume of the droplet. The total volume of the droplet was divided by the volume of the bulk in the imaged region to obtain the volume fraction. Relative enrichment of tagged protein and labeled poly-rU was measured from the mean fluorescent intensity protein and poly-rU inside PGL-3/RNA condensate divided with the mean fluorescent intensity in the bulk. Codes are available on request.

### Statistics.

Data was analyzed and exported using R (1.1.447). t test was used to compare means between samples. Significance levels are *P < 0.5, **P < 0.01, and ***P < 0.001

### Microfluidic Device Design and Fabrication.

Devices for phase diagram measurements (Fig. 4) were fabricated as previously described (29). Briefly, three and four inlet devices were designed with AutoCAD (AutoDesk) and printed on a photomask (Micro Lithography). On an SU8-3050 photoresist (Microchem) coated (50 μm) silicon wafer, the structure was engraved by UV exposure (40 s). After removal of excessive photoresist with propylene glycol methyl ether acetate (Sigma Aldrich), the coated and imprinted wafer was baked (1 min, 95 °C). Then, it was put into a petri dish and a 10:1 mixture of poly(dimethylsiloxane) (Sylgard 184, Dow Corning) and crosslinking agent was poured on top. Air was removed under vacuum (1 h), and subsequently, the dish was baked (65 °C, 1 h). Using a scalpel, the device was cut and inlet holes were added. A clean glass slide and the device were activated in an oxygen plasma oven (30 s, 60% power, Femto, Diener Electronics) and bonded. Before employment on the microfluidic platform, the microfluidics channels were treated with trichloro(1H,1H,2H,2H-perfluorooctyl) silane (1% v/v, Sigma Aldrich) in HFE-7500 (Fluorochem) and heated (95 °C, 30 min).

### Phase Diagram Acquisition and Imaging.

Stock solutions of PGL-3::His6::mEFP, MBP::MEX-5::tagRFP, MBP::MEX-5∆ZF::tagRFP, and poly-rU were prepared in 200 mM KCl (Invitrogen). RNA was barcoded with AF647 (Thermo Fisher Scientific). The device was connected to an HFE-7500/008-FluoroSurfactant (RAN Biotechnologies) mixture, buffer (25 mM HEPES pH 7.3, 200 mM KCl, and 1 mM DTT), and the protein/RNA stock solutions. To reach a wide range of concentrations, the component flow rates were tuned by pressure pumps (Fluigent). Water-in-oil droplets were made on chip (Fig. 3). In an imaging chamber, they were imaged every 5 s by means of an epifluorescence microscope (Cairn Research) equipped with a 10x objective (Nikon CFI Plan Fluor 10×, NA 0.3).

Analysis of the PhaseScan images was carried out by means of a custom-written Python script. Droplets and PGL-3/RNA condensates were detected by a trained convolutional neural network. Comparison of the fluorescence intensities of the recorded images to the calibration images allowed to convert into concentrations. The dilute phase concentration of PGL-3 was determined through the darkest 5 to 25% pixels inside droplets for tie line and dominance evaluation.

### Microfluidic Diffusional Sizing.

Samples were prepared at 1 μM protein and increasing poly-rU (0 to 6,800 ng μL^−1^), followed by incubation (15 min) and subsequent centrifugation in order to separate the condensed phase from the supernatant. Prepared samples were incubated for 15 min and subsequently centrifuged (5 min, 15,000 rpm, Eppendorf 5424 R) to diminish the number of RNA/PGL-3 condensates in solution. Then, buffer (25 mM HEPES pH 7.3, 200 mM KCl, and 1 mM DTT) and samples were added on chip (Fluidic Analytics) and measured on the Fluidity One-M instrument (Fluidic Analytics) at standard settings (488 nm, size range 3 to 14 nm) (Fig. 4). Curve fitting to extract the *K*_d_ was performed as described in the literature (52, 53).

### BN-PAGE.

5 μg of phosphorylated MEX-5 diluted in sample buffer to a final concentration of 50 mM BisTris, 6 N HCl, 50 mM NaCl, 10% w/v glycerol, and 0.001% Ponceau S, pH 7.2, were loaded onto NativePAGETM 4 to 16%, Bis-Tris (ThermoFisher Scientific, BN1002BOX). 1× NativePAGE TM Running Buffer (ThermoFisher Scientific, BN2001) was used for anode buffer and NativePAGETM Running Buffer was combined with NativePAGETM Cathode Additive (ThermoFisher Scientific, BN2002) to make 1x Cathode buffer containing a final concentration of 0.002% G-250 Coomassie. The gels were run at a constant voltage of 150 V for 2 h, washed with deionized water, fixed with 40% methanol, 10% acetic acid, stained with 0.02% Coomassie R-250 in 30% methanol, and 10% acetic acid.

### Poly-rU Labeling.

Mix 40 μg of poly-rU, 1X T4 RNA buffer (NEB), 10% V/V DMSO, 1 mM ATP, 40 μM pCp-Cy5 (Jena BioScience), and 10 U T4 RNA Ligase 1 (NEB). Incubate reaction at 16 °C overnight. Heat reaction to 65 °C for 10 min to inactivate the ligase. Purify the reaction using Spin Columns (Sigma). Precipitate overnight in EtOH at −20 °C followed by washing in 75% EtOH and resuspension in nuclease-free water.

## Supplementary Material

Appendix 01 (PDF)

Movie S1. MEX-5-mEGFP and PGL-3-mCherry are dynamic during the Oocyte to Embryo transition (OET) in *C. elegans*. Both PGL-3-mCherry condensates and cytosolic MEX-5- mEGFP are enriched in the oocytes. MEX-5-mEGFP appears to increase as oocytes mature. During OET, PGL-3-mCherry disassembles as MEX-5-mEGFP is uniformly distributed. On polarization, MEX-5-mEGFP and PGL-3mCherry enrich in the anterior half and the posterior half respectively of the one-cell embryo before the first cell division.

Movie S2. RNA digestion coarsens perinuclear PGL-3-mEGFP in vivo as disassembly progresses after RNaseA injection in the gonad of *C. elegans.* Maximum intensity projections of confocal z-slices of PGL-3-mEGFP in adult worm germline syncytium is acquired from 15 to 90 minutes after 0.5 μg/ml RNaseA injection. PGL-3-mEGFP condensates coalesce and disassemble from 15 to 60 minutes after RNaseA injection (0.5 μg/ml). (n=3)

## Data Availability

Figures raw data has been deposited on the platform Zenodo under DOI: 10.5281/zenodo.12193456 (54).
