# Supplementary material for: A mechanism for MEX-5-driven disassembly of PGL-3/RNA condensates in vitro
Source: Proc Natl Acad Sci U S A. 2025 May 12;122(20):e2412218122. doi: 10.1073/pnas.2412218122 (PMC12107180; doi:10.1073/pnas.2412218122)
Supplement: Supplementary file 1 — Appendix 01 (PDF) [file pnas.2412218122.sapp.pdf]

PNAS

Supporting Information for
A mechanism for MEX-5-driven disassembly of PGL-3/RNA condensates in vitro.

Natasha S. Lewis, Silja Zedlitz, Hannes Ausserwöger, Patrick McCall, Lars Hubatsch, Marco Nousch, Martine Ruer-Gruß, Carsten Hoege, Frank Jülicher, Christian R. Eckmann, Tuomas Knowles, Anthony A. Hyman

Anthony A. Hyman
Email: hyman@mpi-cbg.de

This PDF file includes:

Supporting Information Text
Figures S1 to S6
Legends for Movies S1 and S2
SI References

Other supporting materials for this manuscript include the following:

Movies 1 to 2

Supporting Information Text:

Comparison to Saha *et al.* 2016[1]: Droplet hardening was shown by Saha *et al.*[1] using total mRNA and was resolved in our work by screening of homopolymeric RNA. In vivo, helicases such as GLH-1 are required to unwind mRNA in the P granules. However, our in vitro assay demonstrates that the presence of long unfolded poly-rU can effectively bypass the need for a helicase.

The purification of full-length MEX-5 has proven to be challenging previously[1], and instead the MEX-5 zinc finger domain 236-350 was used for the in vitro assay[1]. In the current work, we achieved solubilisation of full-length MEX-5 by tagging with MBP at the N-terminus. Further, we purified from insect cells while Saha *et al.*[1] used E.coli. As described by Pagano *et al.* 2007[2], zinc was added in both protocols in the early steps during purification, however in our work it was not included in the final elution buffer. Tavella *et al.* 2020[3] indicated that the zinc finger domain is further stabilized upon binding to RNA, hence to reduce protein aggregation, we thawed fresh protein and filtered it before use, kept MEX-5 on ice and introduced it directly into our assay containing RNA, ensuring that excess zinc is not present in the solution. This method effectively preserved the stability and functionality of MEX-5 during purification.

Comparison to Smith et al. 2016[4]: A key distinction is that Smith et al. 2016[4] examined MEG-3, whereas our work focuses on PGL-3. MEG-3 is largely disordered, while PGL-3 contains a globular domain. Smith *et al.*[4] used for their in vitro model MEG-3 and poly-rU30 and showed how MEX-5 inhibits RNA-induced phase separation while our work focuses on the effect of MEX-5 on PGL-3/RNA condensates. The binding affinities of the proteins to RNA (32[4]/744[5] nM for MEG-3 and 3.4 μM for PGL-3) differ significantly. Further, PGL-3/RNA condensate formation has been shown to be efficient with a minimal RNA length of ~500 bases (Saha *et al.* 2016[1]) which is considerably longer as compared to the 30 bases long poly-rU used with MEG-30 by Smith *et al.*[4]. Together, these findings make direct comparisons between the systems challenging, also taking into account the distinct material properties and diffusion coefficients for the purified proteins (Folkmann *et al.* 2021[6]).

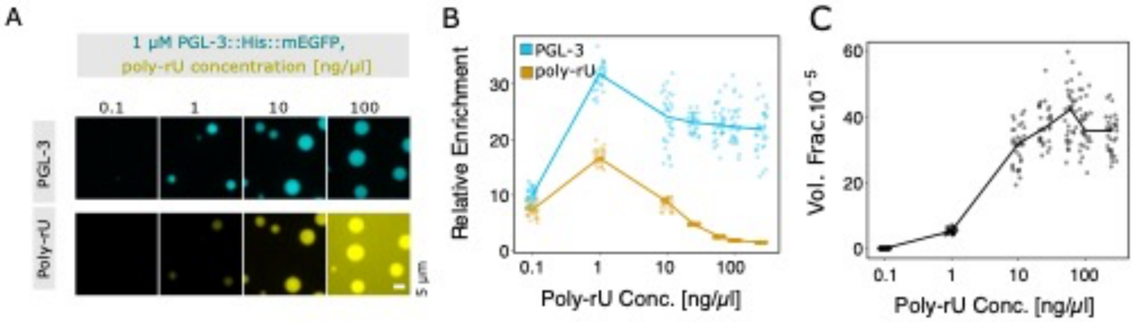

Fig. S1. (A) Maximum intensity projections of confocal z-slices of PGL-3-6xHis-mEGFP (1 µM) condensates with labelled poly-rU (0.1, 1, 10, 100 ng/µl) were acquired 1 hour after inducing assembly by lowering salt concentration. Both the size of the condensate and the intensity of labelled poly-rU in the assay appears to increase. (B) The relative enrichment of both PGL-3 and poly-rU inside condensates increases in poly-rU followed by a decrease in the enrichment. (C) The volume fraction (Vol. Frac.) of condensates increases in poly-rU followed by a decrease in volume fraction. (n=3).

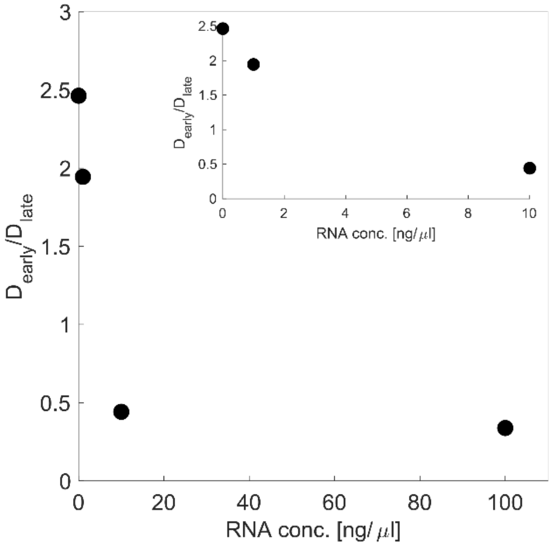

Fig. S2. Ratio of diffusion coefficients of PGL-3/RNA condensates over time. FRAP data was recorded after 0.5 and 3 h post condensate formation at 22 °C. For every condition n = 2.

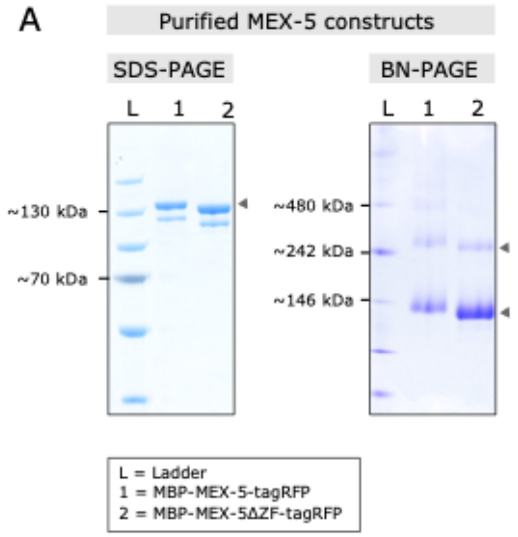

Fig. S3. Purified MEX-5 proteins. (A) SDS-PAGE of purified MBP-MEX-5-tagRFP and MBP-MEX-5ΔZF-tagRFP. The annotated brighter band is the purified protein constructs, dimmer band is tag-RFP hydrolysed from MS. BN-PAGE of purified MBP-MEX-5-tagRFP and MBP-MEX-5ΔZF-tagRFP. The annotated brighter band is the monomeric fraction of the native purified protein constructs, dimmer band is the dimeric fraction of the native purified protein constructs.

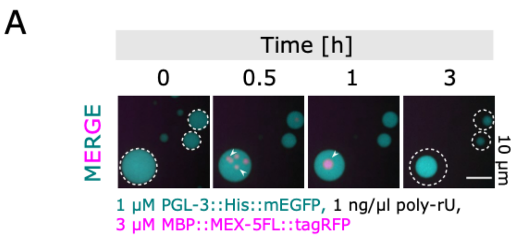

Fig. S4. Nested MBP-MEX-5-tagRFP drops inside condensates. (A) Maximum intensity projections of confocal z-slices of PGL-3-6xHis-mEGFP (1 µM) condensates with poly-rU (1 ng/µl) were acquired 0,0.5,1,3 hours after adding MBP-MEX-5-tagRF at 3 µM. Nested MBP-MEX-5-tagRFP drops annotated with arrows are seen entering condensates, fusing, and exiting condensates. The dotted circle marks the periphery of condensates to track the decrease in size of the condensate.

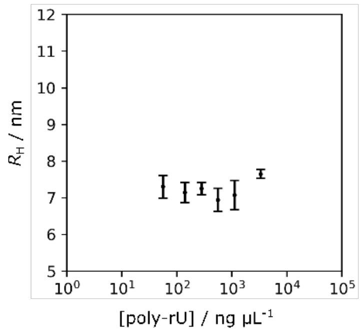

Fig. S5. Binding affinity of MEX-5ΔZF to poly-rU via microfluidic diffusional sizing. MEX-5ΔZF shows no binding to poly-rU RNA. Fluctuations are within error (standard deviation).

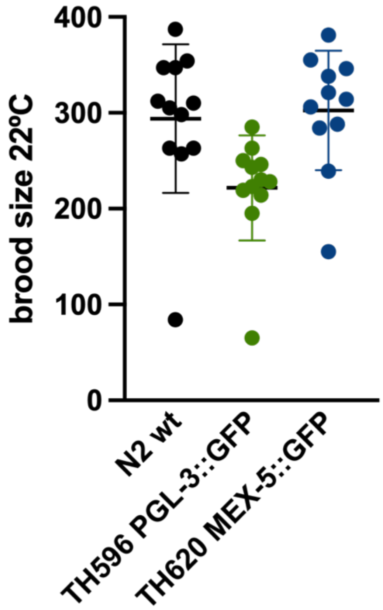

Fig. S6. Effect of protein tagging on brood size and in vivo fertility. Brood sizes of *C.elegans* wild type and endogenously tagged PGL-3::mEGFP and MEX-5::mEGFP worms at 22 ºC with fertility rates remaining near 100% for wild-type and PGL-3::mEGFP, and 92% for MEX-5::mEGFP.

Movie S1. MEX-5-mEGFP and PGL-3-mCherry are dynamic during the Oocyte to Embryo transition (OET) in *C. elegans*. Both PGL-3-mCherry condensates and cytosolic MEX-5-mEGFP are enriched in the oocytes. MEX-5-mEGFP appears to increase as oocytes mature. During OET, PGL-3-mCherry disassembles as MEX-5-mEGFP is uniformly distributed. On polarization, MEX-5-mEGFP and PGL-3mCherry enrich in the anterior half and the posterior half respectively of the one-cell embryo before the first cell division.

Movie S2. RNA digestion coarsens perinuclear PGL-3-mEGFP in vivo as disassembly progresses after RNaseA injection in the gonad of *C. elegans*. Maximum intensity projections of confocal z-slices of PGL-3-mEGFP in adult worm germline syncytium is acquired from 15 to 90 minutes after 0.5 μ/ml RNaseA injection. PGL-3-mEGFP condensates coalesce and disassemble from 15 to 60 minutes after RNaseA injection (0.5 μg/ml). (n=3)

SI References:

1. Saha, Shambaditya et al. "Polar positioning of phase-separated liquid compartments in cells regulated by an mRNA competition mechanism." *Cell* 166.6 (2016): 1572-1584.
2. Pagano, J. M. et al. “Molecular basis of RNA recognition by the embryonic polarity determinant MEX-5.” *Journal of Biological Chemistry*, 282.12 (2007): 8883–8894.
3. Tavella, Davide et al. "A disorder-to-order transition mediates RNA binding of the Caenorhabditis elegans protein MEX-5." *Biophysical Journal* 118.8 (2020): 2001-2014.
4. Smith, Jarrett et al. "Spatial patterning of P granules by RNA-induced phase separation of the intrinsically-disordered protein MEG-3." *Elife* 5 (2016): e21337.
5. Schmidt, Helen et al. "Protein-based condensation mechanisms drive the assembly of RNA-rich P granules." *Elife* 10 (2021): e63698.
6. Folkmann, Andrew W. et al. "Regulation of biomolecular condensates by interfacial protein clusters." *Science* 373.6560 (2021): 1218-1224.
